# Acknowledgement to Reviewers of *Brain Sciences* in 2013

**DOI:** 10.3390/brainsci4010123

**Published:** 2014-03-03

**Authors:** Brain Sciences Editorial Office

**Affiliations:** MDPI AG, Klybeckstrasse 64, CH-4057 Basel, Switzerland

The editors of *Brain Sciences* would like to express their sincere gratitude to the following reviewers for assessing manuscripts in 2013:
Abizaid, AlfonsoGommer, ErikNedergaard, MaikenAgrawal, Sandeep K.Gonzalez-Lima, FranciscoNenadic, IgorAhn, EdGorczynski, PaulNickels, LyndseyAlbuquerque, FelipeGriffin, ZenziNimmo, AlanAlkayed, NabilGruol, DonnaNovelli, LuanaArmstrong, ReginaGuizzetti, MarinaOechslin, Mathias S.Aton, SaraGvilia, IrmaOstrowski, RobertAugustenborg, ClaudiaHagenauer, MeganParnell, ScottBahramisharif, AliHasan, Bashar Awwad ShiekhPires, GabrielBai, OuHeape, AnthonyQiang, MeiBallard, KirrieHemmen, Thomas M.Quinlan, Katharina ABallard, MarkHirotani, MasakoRaizen, DavidBansal, VishalHochman, DarylRapp, DavidBel, FrankHoffman, PaulReale, MarcellaBenson, D.l.Hoffman, RalphReinhard, MatthiasBerryhill, MarianHogben, JohnRichlan, FabioBhambhani, YageshHuang, SuiRiikonen, RailiBialystok, EllenHummel, ThomasRobbins, Matthew SBieberich, ErhardHung, Jan-jongRobertson, CoutneyBigler, ErinHutchinson, KentRomero, JulianBirbaumer, NielsIkonomidoua, ChrysanthyRothermich, KathrinBoullerne, AnneJacoby, JacobSammler, DanielaBowden, HarrietJarrott, BevynSavage, DanielBramlett, Helen M.Jessen, KristjanSayeed, IqbalBraun, KatharinaJevtovic-Todorovic, VesnaScafidi, JosephBritz, GavinJiang, LijunSchenck, CarlosBroussalis, ErasmiaJoubert, SvenSchengrund, Cara-lynneBrouwer, Anne-MarieKaan, EdithScott, OlsonBruno, GuglielmoKansaku, KenjiSerafini, SandraBrysbaert, MarcKaur, CharanjitShaul, ShelleyCavazza, MarcKaushanskaya, MargaritaShaw, PaulChai, Xiaoqian JKeyser, JacquesShohami, EstherChalak, Lina F.Khalessi, AlexanderShuaib, AshfaqChampagne, Frances A.Kim, HelenaSims, Neil R.Chau, TomKooijman, RonSlevc, L. RobertChilibeck, PhilKousaie, ShannaSoriano, Sulpicio G.Chrissobolis, SophoclesKroener, SvenSourina, OlgaCirelli, ChiaraKrusienski, Dean J.Spengler, DietmarCollins, M. A.Kuperberg, GinaSteinman, LawrenceColrain, IanKurth, SalomeStone, Trevor W.Cook, Stuart D.Le, Anh DzungSumbria, Rachita K.Cox, JessicaLeybaert, JacquelineTervaniemi, MariCrepaldi, DavideLi, XueyiTiwari-Woodruff, SeemaCunha, RodrigoLight, KimTobin, YishaiD'Ausilio, AlessandroLipinski, Christopher A.Tsunoda, IkuoDaadi, Marcel M.Liu, DazhiUchikoshi, YuukoDalrymple-Alford, JohnLonstein, JosephValdez, R. ChavezDasgupta, SomsankarLooney, DavidVan Dyke, Julie A.Desai, RutvikLu, PaulVan Os, JimDeutsch, DiannaLuo, JiaVancampfort, DavyDiaz, Michele T.Ma, DaqingVercammen, AnsDing, NaiMagliano, JosephWambaugh, Julie L.Doesburg, SamMajid, ArshadWang, XinDuffau, H.Manzanero, SilviaWardi, TaraDuñabeitia, Jon AndoniMarinova-Todd, Stefka H.Warrington, ElizabethEl Idrissi, AbdeslemMccaffery, PeterWenning, LeslieElliott, Melanie B.Mclaughlin, MarkWilliamson, VickyEstevez, AnaMeltzer, JedWilling, Alison E.Fainsod, AbrahamMéndez Zorrilla, AmaiaWitton, CarolineFaraguna, UgoMiano, SilviaWoollams, AnnaFatemi, S. AliMiranda, RajeshXie, ZCFernández-Ruiz, JavierMizuo, KXu, XinFielder, GraceMorone, GiovanniYalachkov, YavorFitch, Roslyn H.Myung, JayYamamoto, AkihitoFleming, Rebekah L.Nagai, AtsushiYazdani, AshkanFutschik, Matthias ENagel, BonnieYoshimoto, KanjiGale, KarenNazarpour, KianoushZiv, Noam

